# The Effect of Thickened Water on Ciprofloxacin Pharmacokinetics: A Comparative Study in Adult Males

**DOI:** 10.3390/jcm13154573

**Published:** 2024-08-05

**Authors:** Iori Taki, Taigi Yamazaki, Nobuyuki Takahashi, Myong Hwa Yamamoto, Akiko Toju, Atsuko Ikeura, Eisuke Inoue, Takehiko Sambe, Takuya Mizukami, Naoki Uchida, Tsutomu Harada, Noriko Hida

**Affiliations:** 1 Department of Clinical Research and Development, Graduate School of Pharmacy, Showa University, Tokyo 157-8577, Japan; t.yamazaki@cmed.showa-u.ac.jp (T.Y.); ikeura.atsuko@med.showa-u.ac.jp (A.I.); n.hida@med.showa-u.ac.jp (N.H.); 2 Showa University Clinical Research Institute for Clinical Pharmacology and Therapeutics, Tokyo 157-8577, Japan; myong@med.showa-u.ac.jp (M.H.Y.); akiko.toju@med.showa-u.ac.jp (A.T.); t-sambe@med.showa-u.ac.jp (T.S.); mizukamit@med.showa-u.ac.jp (T.M.); nuchida@med.showa-u.ac.jp (N.U.); 3 Department of Hospital Pharmaceutics, School of Pharmacy, Showa University, Tokyo 142-8555, Japan; t.nobuyuki@cmed.showa-u.ac.jp; 4 Research Administration Center, Showa University, Tokyo 142-8555, Japan; eisuke.inoue@med.showa-u.ac.jp; 5 Department of Clinical Pharmacology, Graduate School of Medicine, Showa University, Tokyo 157-8577, Japan; 6 Department of Pharmaceutics, Graduate School of Pharmacy, Showa University, Tokyo 157-8577, Japan; tharada@pharm.showa-u.ac.jp

**Keywords:** dysphagia, ciprofloxacin, food thickener, xanthan gum, healthy adult male, pharmacokinetics, food interaction, dysphagia

## Abstract

**Background/Objectives**: The use of food thickeners with ciprofloxacin tablets may result in a gelatinous appearance and experience delayed dissolution, which presents a challenge for the drug’s efficacy, creating a healthcare economic issue. However, the pharmacokinetic impact of this compound in humans remains uncertain. Therefore, a comparative pharmacokinetic study of ciprofloxacin was conducted on healthy adult Japanese males. **Methods**: We compared the effects of administering tablets with water or thickened water and crushed tablets mixed with thickened water. The maximum blood concentration (C_max_) of ciprofloxacin determines the drug’s efficacy. **Results**: There were variations in drug absorption across different administration methods. The group who took the tablets immersed in thickened water exhibited different results in the area under the blood drug concentration–time curve (AUC) and C_max_ compared to the group who took the tablets in regular water. Notably, the group that consumed the crushed tablets mixed with thickened water demonstrated equivalent results for both AUC and C_max_. **Conclusions**: Administering crushed tablets in thickened water may yield pharmacokinetics comparable to those of tablets taken with water. However, the process of crushing tablets may result in the loss of active ingredients and compromise the formulation, necessitating a comprehensive assessment before administration.

## 1. Introduction

The risk of aspiration due to dysphagia increases with age and the presence of underlying medical conditions [1,2]. A systematic review by Rajati et al. [3] estimated the global prevalence of dysphagia to be 43.8%, with a high prevalence observed in various populations worldwide. This prevalence has recently increased.

Dysphagia can precipitate a range of life-threatening complications, including malnutrition, dehydration, and pneumonia resulting from aspiration. A variety of measures can be employed to address this issue; however, modifying food forms represents a particularly beneficial approach [4]. Thickeners are available in powdered form for thickening existing liquids and foods, thus facilitating the process of swallowing. Thickening agents are formulated with starch, processed starch, or xanthan gum as the primary ingredient. Previously, thickening agents based on starch or processed starch have been utilized; however, these have been identified as problematic due to the oral residue which can cause aspiration [4,5], be degraded by amylase [6,7], and be less acceptable to patients [8]. Xanthan gum is attracting attention as a new thickening agent that solves these problems [9].

Xanthan gum is a polysaccharide secreted by the microorganism Xanthomonas campestris, which utilizes sugar as a nutrient source. It is a polysaccharide composed of a repeating unit with two molecules of glucose as the main chain and two molecules of mannose and one molecule of glucuronic acid as the side chains. Due to the length of its side chains, it exhibits remarkable stability. It is a water-soluble polymer with negatively charged carboxyl groups and pyruvic acid in its side chains, and exhibits high viscosity in small amounts [10]. Additionally, it is straightforward to handle as it does not impair the taste, color, or flavor of foodstuffs. Furthermore, it has been shown to be safe as a food additive [11] and is widely used in a variety of foods and thickeners.

Conversely, the use of thickened foods has recently been linked to the disruption of the disintegration and dissolution processes of certain medications. A systematic review of the effects of thickeners on drug bioavailability indicated that the disintegration and dissolution processes appear to be related to the type of thickener, the viscosity of the thickened liquid, the pH, and the soaking time [12]. In a human study, Tomita et al. [13,14] reported that the hypoglycemic effects of two hypoglycemic drugs were affected when tablets were immersed in a food thickener. However, the blood concentration of levofloxacin in the bloodstream remained unaltered [15]. Even when disintegration and delayed dissolution are observed in vitro, the translation to human pharmacology and pharmacokinetic (PK) parameters is not always straightforward.

Patients with dysphagia may be taking a variety of medications for other medical conditions. Caution should be exercised because dosage forms may be altered when medications are crushed or administered mixed with swallowing aids to account for potential problems associated with swallowing oral medications [16], but there is currently no information available for clinical staff to determine these.

Co-authors Takahashi et al. [17] observed that the dissolution of ciprofloxacin tablets in their crushed form was delayed, and the gel-like appearance of the tablets was altered when they were immersed in xanthan gum solution. Nevertheless, it remains uncertain whether this alteration inevitably influences pharmacokinetics in humans. Consequently, the authors recommend that it be circumvented in humans for the time being.

Ciprofloxacin is classified as a new quinolone antibacterial agent. It exhibits broad-spectrum antibacterial activity and relatively few side effects, and is a drug that has been used worldwide for many years, making it a highly useful drug [18,19]. It is typically employed as a last resort to treat antibiotic-resistant infections [19]. However, the emergence of resistant bacteria in response to ciprofloxacin has also posed a challenge [20]. Given that quinolone antibacterial agents are concentration-dependent drugs [21] and that blood levels influence both drug efficacy and the development of bacterial resistance, it was deemed essential to examine the PK profile of ciprofloxacin in humans when administered with food thickeners. The use of food thickeners has the potential to impair the desired therapeutic outcomes and create problems from a healthcare economic perspective due to decreased absorption [22].

Ciprofloxacin is coated with a film to mask its bitter taste, but it is also anticipated that it will be crushed when taken with thickened foods or administered via a tube. The objective of this study was to investigate and evaluate the impact of xanthan gum-based thickened water (drinking water thickened with food thickener) on the PKs of ciprofloxacin tablets and crushed ciprofloxacin tablets, as compared to the PKs of ciprofloxacin tablets when taken with ordinary drinking water.

## 2. Materials and Methods

### 2.1. Study Participants

This study included healthy adult males between the ages of 20 and 45 years who were capable of providing informed consent and who complied with the study’s rules of compliance during participation. The participants were required to undergo a preliminary examination and report any subjective symptoms as stipulated in the study protocol. Individuals were excluded if they were taking any medications (including dietary supplements), had a history of drug allergies, were participating in other clinical studies within three months, or were deemed ineligible by the investigators.

All participants were provided with comprehensive information about the present study and requested to provide written consent.

### 2.2. Study Drugs

Ciproxan^®^ Tablets 200 mg (each tablet contains 232.8 mg of ciprofloxacin hydrochloride hydrate, Lot No. JPS5899; Bayer Yakuhin, Ltd., Osaka, Japan) were administered. The thickener utilized in this study was Tsururinko^®^ Quickly (dextrin/xanthan gum, calcium lactate, trisodium citrate (content unknown); Clinico Corporation, Tokyo, Japan), following the methodology by Takahashi et al. This thickening control food contains xanthan gum as its primary ingredient and is commercially available and commonly used in Japan.

### 2.3. Study Design

**Figure 1:**
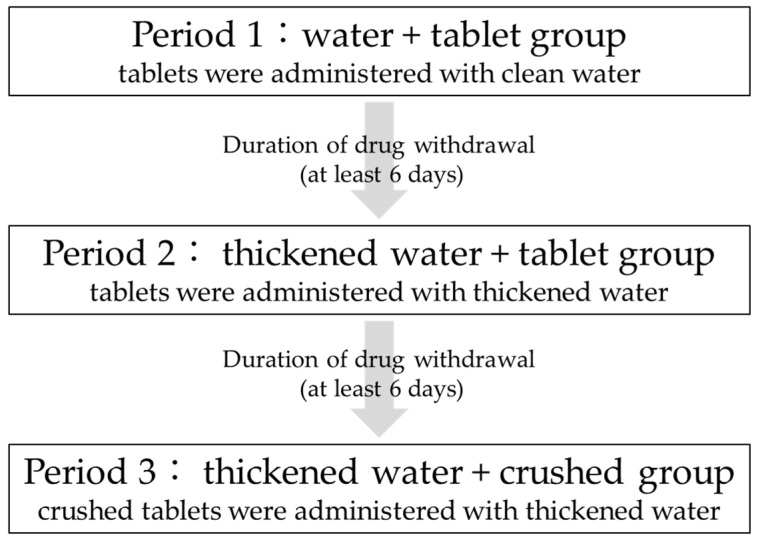
Schedule of entire study.

This study was a three-arm, three-period, open-label study in which the tablets were administered with clean water, then thickened water, and finally crushed in thickened water, all administered to the same participants. Figure 1 shows the drug administration schedule. All participants were admitted to the hospital the day before administration and discharged after 24 h of blood sampling and examination. A six-day washout period was observed after drug administration in each period.

### 2.4. Administration Method

#### 2.4.1. Adjustments of Thickened Water

**Figure 2:**
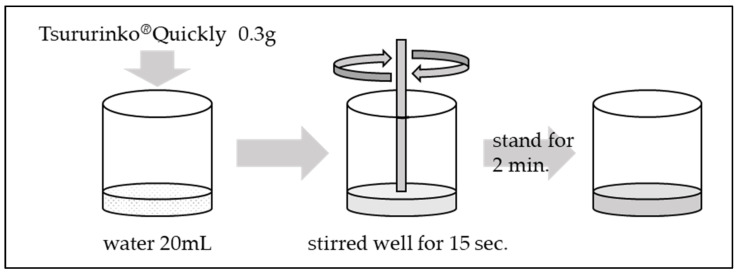
Adjustment of thickened water.

The methodology for adjusting thickened water is illustrated in Figure 2.

The standard concentrations listed in the product outline for Tsururinko^®^ Quickly are 1% (designated as “thin thickening”), 2% (designated as “intermediate thickening”), and 3% (designated as “thickening”). The concentration employed in the present study was 1.5%, which was previously utilized by Takahashi et al.

In a container containing 20 mL of water, 0.3 g of Tsururinko^®^ Quickly was added and stirred well for 15 s. Subsequently, the final product was then allowed to stand for a period of 2 min.

#### 2.4.2. Administration Method

The study participants were administered a dose of 200 mg ciprofloxacin.

On the day preceding the administration of the drug, the participants were instructed to refrain from consuming food or water after dinner. One hour prior to administration, the participants abstained from drinking water. Subsequent to the administration of the drug, the participants were directed to adhere to a period of fasting and bed rest for a duration of 4 h.

**Figure 3:**
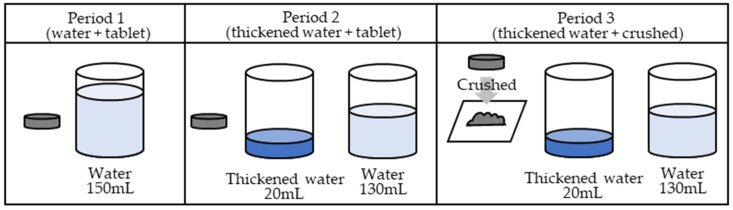
Administration method.

In the first period, the ciprofloxacin tablets were orally administered with 150 mL of water. In the second period, the ciprofloxacin tablets were soaked in 20 mL of thickened water and administered orally, immediately followed by 130 mL of water. In the third period, a ciprofloxacin tablet was placed between two sheets of wrapping paper and crushed uniformly with a pestle on top of the wrapping paper. Upon crushing, the tablets were visually confirmed to have been crushed uniformly. Figure 3 illustrates the procedure.

In period 1 (water + tablet group), a 200 mg ciprofloxacin tablet was orally administered with water. In period 2 (thickened water + tablet group), a 200 mg ciprofloxacin tablet was orally administered as a tablet with 20 mL of thickened water, and an additional 130 mL of water was additionally consumed. In period 3 (thickened water + crushed group), 200 mg of the crushed ciprofloxacin tablet was orally administered with 20 mL of thickened water, and 130 mL of water was additionally consumed. A six-day withdrawal period was permitted between periods 1 and 2 and between periods 2 and 3to allow for the metabolic elimination period.

### 2.5. Blood Sampling

Blood samples were collected at 10 time points: prior to administration and at 0.5, 1.0, 1.5, 2, 4, 6, 8, 12, and 24 h post-administration. Each sample consisted of 5 mL of blood collected from the median cutaneous vein. The objective was to measure blood levels. The blood drug concentrations were assessed in accordance with the study schedule and are presented in Table 1.

### 2.6. Measurement of Blood Drug Concentration

The plasma was subjected to centrifugation at 4 °C for 10 min at 3000 rpm. Thereafter, it was frozen at or below −80 °C until the concentration of the drug could be analyzed. Subsequently, the separated plasma samples were dispatched to Japan Medical Laboratory, Inc., for measurement using liquid chromatography–tandem mass spectrometry (LC-MS-MS) methods. The measurement conditions were as follows.

The liquid chromatography (LC) was performed using the LC-30A system (Shimadzu Corp., Kyoto, Japan), while the mass spectrometry (MS-MS) was conducted with the Triple Quad 6500+ system (AB Sciex Pte. Ltd., Shinagawa, Tokyo, Japan). The analytical column was Cadenza CD-C18 (50 mm length, 4.6 mm inner diameter and 3 μm particles, Intact Corp., Kyoto, Japan). The mobile phase was a linear gradient of 5 mmol/L ammonium formate containing 0.2% formic acid and acetonitrile. The flow rate was set at 0.3 mL/min and the column temperature was maintained at 40 °C. The LC-MS/MS system parameters were set as follows: ion spray voltage, 4500 V; temperature, 500 °C; curtain gas, 40 (nitrogen); ion source gas 1, 30 (zero grade air); ion source gas 2, 40 (zero grade air).

In order to achieve ionization, positive ion detection (multiple reaction monitoring, MRM) was employed in the electrospray ionization method (Turbo Spray), resulting in the detection of CPFX (m/z 332.0 → 231.0). The data were acquired using Analyst software, version 1.6.3 (AB Sciex Pte. Ltd., Shinagawa, Tokyo, Japan).

The lower limit of detection was established at 0.01 μg/mL. ISR measurements were conducted for a single blood concentration point per participant in each period, with 100% determined.

### 2.7. Calculation of PK Parameters

The primary PK parameters calculated included the area under the blood drug concentration–time curve (AUC_0→24_) and the maximum blood concentration (C_max_) up to 24 h after administration. Secondary endpoints included the time to maximum blood concentration (t_max_) and disappearance half-life (t_1/2_).

### 2.8. Statistical Analysis

Subsequent analyses were conducted on the basis of the fixed data obtained from the evaluation. In the PK and bioequivalence analyses, only cases that had been completed were included, while discontinued cases were excluded. The blood drug concentrations from the discontinued and excluded cases were documented up to the time of discontinuation. All patients who received the study drug were included in the safety evaluation.

Efficacy and safety analyses were conducted, and the equivalence of PK parameters was explored in comparison with the first period. The background of the study population was included in the efficacy analysis, and summary statistics were calculated for background factors.

For the primary endpoint, repeated-measures analysis of variance models were employed to analyze the two-sided 90% and 80% confidence intervals for the difference between the means of AUC_0→24_ and C_max_ in periods 1 and 2 and periods 1 and 3. The equivalence of AUC_0→24_ was determined using two-sided 90% confidence intervals, with equivalence defined as values falling between −30% and 30% when divided by the mean value of one period. The equivalence of C_max_ was determined using two-sided 80% confidence intervals, with equivalence defined as values falling between −40% and 40% when divided by the mean value of one period. Similarly, a repeated-measures analysis of variance model was employed to analyze the secondary endpoints, calculating the mean values and 95% confidence intervals for t_1/2_ and t_max_ between periods 1 and 2 and periods 1 and 3. R (ver. 4.3.0) was used for the analysis.

### 2.9. Ethical Considerations

This study was conducted in accordance with the Declaration of Helsinki, the Ethical Guidelines for Medical Research Involving Human Participants, and the Clinical Research Act. This study was approved by the Institutional Review Board of the Showa University Clinical Research Review Board (jRCTs031220436).

## 3. Results

### 3.1. Participants

A total of six participants were included in this study. The participants had a mean age of 28.50 ± 8.46 years, height of 172.43 ± 4.77 cm, weight of 64.72 ± 4.84 kg, and body mass index of 21.77 ± 1.69. All participants completed the study, without discontinuation or dropout, thus satisfying the inclusion criteria for the efficacy and safety analysis.

### 3.2. PK Parameters

**Figure 4:**
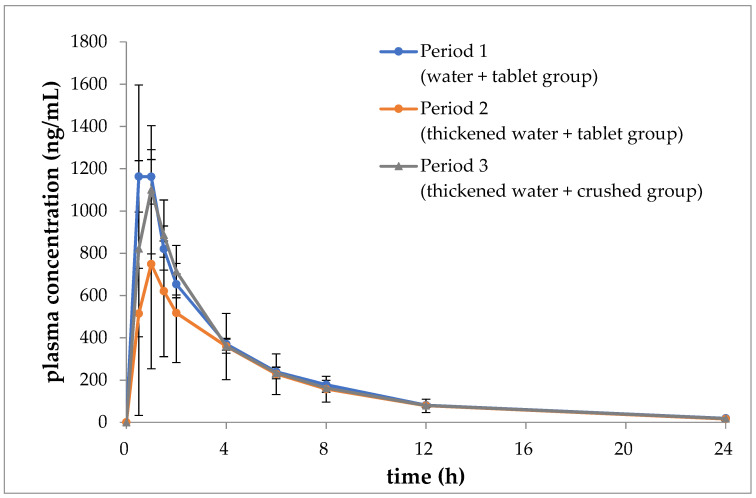
Blood concentrations of ciprofloxacin with time (mean ± SD, *n* = 6).

Figure 4 depicts the temporal trends in the mean blood concentrations of ciprofloxacin during periods 1, 2, and 3. Table 2, Table 3 and Table 4 present the mean values of AUC_0→24_, C_max_, t_max_, and t_1/2_ for each period, along with the corresponding analysis results.

The two-sided 90% confidence interval (−2073.34 to 139.34) for the difference between the mean of AUC_0→24_ in period 2 and period 1 was found to range from −42% to 3%. This range was found to fall outside the predefined lower limit range of −30% to 30%. Similarly, the two-sided 80% confidence interval (−753.41 to −208.09) for the difference between the mean of C_max_ in the first period and divided by the mean of the first period was found to be from −57% to 16%. This interval was found to deviate from the lower limit of −40% to 40%. It can be concluded that there was no equivalence demonstrated in AUC_0→24_ and C_max_ between the second and first periods.

However, the two-sided 90% confidence interval (−1291.42 to 921.26) for the difference between the mean of AUC_0→24_ in periods 3 and 1, divided by the mean of period 1, was found to range from −26% to 19%. This interval was calculated using a two-sided 90% confidence interval, with a lower limit of −1291.42 and an upper limit of 921.26. This range fell within the 80% confidence interval for equivalence, which is defined as a range of ±30%. Similarly, the two-sided 80% confidence interval for the difference between the mean C_max_ in the first period, divided by the mean of the first period, was calculated to be from −32% to 9%. This interval fell within the 40% to −40% equivalence range. Therefore, equivalence was demonstrated between the third and first periods with regard to both AUC_0→24_ and C_max_.

The t_max_ and t_1/2_ values were comparable in either the second or third period in comparison to the first period.

### 3.3. Safety Assessment

No adverse events were observed throughout the study period. There were no abnormalities reported in subjective symptoms or other findings.

## 4. Discussion

The use of thickened foods is of paramount importance for the prevention of aspiration [23,24], which makes it challenging to impose limitations on their use. It is of the utmost importance that healthcare providers and caregivers are aware that the use of food thickeners may potentially compromise the effectiveness of medications [25,26]. We hypothesized that if soaking crushed ciprofloxacin tablets in thickened water changes their appearance to be gel-like and slows their dissolution, as shown in the results of the study by Takahashi et al. [17], it may also affect their effect on human PKs.

In this study, we observed that tablet absorption was reduced when immersed in thickened water and when crushed and mixed, indicating that immersion does not necessarily affect human PKs, even with a gel-like change. The results indicate that the collapse process may have been rate-limiting. Ciprofloxacin hydrochloride is a Biopharmaceutics Classification System (BCS) Class IV compound with low solubility and membrane permeability [19]. Furthermore, it is a basic compound, that is, its solubility is high under acidic conditions and decreases as it approaches neutrality [19]. For a medicinal product to be absorbed, the dosage form must disintegrate and the active ingredient must elute before reaching the absorption site. For ciprofloxacin to be absorbed by the body, it must have disintegrated when it reaches the upper gastrointestinal tract, which is under acidic conditions. Despite the gel-like appearance change, the dissolution process appeared to be unaltered, as evidenced by the comparable Cmax and AUC values between the first period and the period where the crushed product was administered. The crushed products were considered to have eluted the components more rapidly because the tablets were forced to disintegrate prior to administration. However, the second period demonstrated reduced lower Cmax and AUC values when the tablets were immersed intact, indicating inadequate elution of components to reach the absorption site. The results of the third period demonstrated the significant impact of the disintegration process. Consequently, it was proposed that the efficacy of pharmaceuticals when consumed with a thickening agent could be enhanced by utilizing dosage forms with smaller particles and larger surface areas, such as crushed tablets or dispersions.

Although ciprofloxacin used in this study is a BCS Class IV drug, even non-Class IV drugs cannot be relied upon. Even highly soluble, highly membrane-permeable Class I drugs and highly soluble, poorly membrane-permeable Class III drugs are known to have reduced bioavailability when used with thickening solutions. Regarding paracetamol tablets, whose main ingredient is acetaminophen (class I), used as an antipyretic analgesic, the experimental results from both in vivo and in vitro studies indicate that increased viscosity may be identified as an important factor causing delayed onset in plasma [25]. The absorption of penicillin (Class III) was found to be significantly reduced when 5 g of guar gum was mixed with 200 mL of water [27]. Solubility and membrane permeability are difficult for nurses and caregivers to determine from package inserts, and it is extremely difficult to determine whether the drug is affected by thickened water when the drug is administered in actual medical practice.

Ciprofloxacin was coated with a film to mask its bitter taste, and crushing was not considered in the formulation design. Consequently, the efficacy of the medication cannot be guaranteed. While crushing could be employed to maintain blood concentrations, any administration method that could compromise the benefits of the formulation design should be meticulously evaluated.

Moreover, the grinding process also resulted in delayed dissolution in the dissolution study conducted by Takahashi et al. [17] under pH 1.2 conditions, which is consistent with previous reports indicating that in vivo results cannot be directly extrapolated to in vitro studies. In vitro disintegration and dissolution studies are conducted to ensure product quality, but the data obtained from these studies do not necessarily reflect PKs in humans. Moreover, the findings of the present study pertain to drugs such as ciprofloxacin which are rapidly absorbed in the upper gastrointestinal tract and may not be generalizable to drugs with a slower absorption rate [28].

It is also important to consider that the impact of thickened foods on pharmaceuticals may be influenced by pH, which can fluctuate in the gastrointestinal tract of humans as a result of disease and dietary habits.

Given the exploratory nature of this study and the limited number of participants involved, further validation, such as increasing the number of participants, is necessary to strengthen the findings.

## Figures and Tables

**Table 1:** Study design.

Elapsed Time (h)	Pre	0	0.5	1	1.5	2	4	6	8	10	12	24
Vital sign measurements	X			X								X
Medical examination	X			X								X
Blood sampling	X		X	X	X	X	X	X	X		X	X
Drug administration		X										
Meal							X *			X		
Subjective symptom survey	X	Any time										
Adverse event observation		Any time										

The X in the table indicates the timing of the inspection. * Meals were permitted once the blood sampling was completed.

**Table 2:** C_max_ of ciprofloxacin in each period.

C_max_ [ng/mL]	Mean [95% CI]	Difference from Period 1 [80% CI]	80% CI/Period 1	
			Lower	Upper
Period 1 (water + tablet group)	1322.83 [1009.76 to 1635.90]	-	-	-
Period 2 (thickened water + tablet group)	842.08 [529.01 to 1155.15]	−480.75 [−753.41 to –208.09]	−0.57	−0.16
Period 3 (thickened water + crushed group)	1172.30 [859.23 to 1485.37]	−150.53 [−423.19 to 122.14]	−0.32	0.09
Criterion			−0.40	0.40

*n* = 6; CI: confidence interval; C_max_: maximum blood concentration. A repeated-measures analysis of variance model was employed to calculate the two-sided 80% confidence intervals for the difference between the means of C_max_ calculated for periods 1 and 2 and periods 1 and 3. Patients were considered equivalent if the two-sided 80% confidence interval divided by the one-period mean value fell between −40% and 40%.

**Table 3:** AUC_0→24_ of ciprofloxacin in each period.

AUC_0→24_ [ng-h/mL]	Mean [95% CI]	Difference from Period 1 [80% CI]	80% CI/Period 1	
			Lower	Upper
Period 1 (water + tablet group)	4895.93 [3934.21 to 5857.65]	-	-	-
Period 2 (thickened water + tablet group)	3928.93 [2967.21 to 4890.65]	−967.00 [−2073.34 to 139.34]	−0.42	0.03
Period 3 (thickened water + crushed group)	4710.85 [3749.13 to 5672.57]	−185.08 [−1291.42 to 921.26]	−0.26	0.19
Criterion			−0.30	0.30

*n* = 6; AUC_0→24_: area under the concentration–time curve; CI: confidence interval. A repeated-measures analysis of variance model was employed to calculate the two-sided 90% confidence intervals for the difference between the means of AUC_0→24_ calculated for periods 1 and 2 and period 1 and 3. Patients were considered equivalent if the two-sided 90% confidence interval divided by the one-period mean value fell between −30% and 30%.

**Table 4:** t_max_ and t_1/2_ of ciprofloxacin in each period.

	t_1/2_ [h]	t_max_ [h]
Period 1 (water + tablet group)	4.95 [4.70 to 5.21]	0.75 [−0.05 to 1.55]
Period 2 (thickened water + tablet group)	4.70 [4.44 to 4.95]	1.42 [0.62 to 2.22]
Period 3 (thickened water + crushed group)	5.02 [4.77 to 5.28]	0.83 [0.03 to 1.63]

*n* = 6; mean and 95% confidence interval; t_max_: time to reach maximum blood concentration; t_1/2_: elimination half-life. The mean values of t_1/2_ and t_max_, along with their respective 95% confidence intervals, were calculated for each period.

## Data Availability

All relevant data are included in the article.

## References

[1] Clavé P., Shaker R. (2015). Dysphagia: Current reality and scope of the problem. Nat. Rev. Gastroenterol. Hepatol..

[2] Baijens L.W., Clavé P., Cras P., Ekberg O., Forster A., Kolb G.F., Leners J.C., Masiero S., Mateos-Nozal J., Ortega O. (2016). European Society for Swallowing Disorders—European Union Geriatric Medicine Society white paper: Oropharyngeal dysphagia as a geriatric syndrome. Clin. Interv. Aging.

[3] Rajati F., Ahmadi N., Naghibzadeh Z.A., Kazeminia M. (2022). The global prevalence of oropharyngeal dysphagia in different populations: A systematic review and meta-analysis. J. Transl. Med..

[4] Clavé P., de Kraa M., Arreola V., Girvent M., Farré R., Palomera E., Serra-Prat M. (2006). The effect of bolus viscosity on swallowing function in neurogenic dysphagia. Aliment. Pharmacol. Ther..

[5] Rofes L., Arreola V., Romea M., Palomera E., Almirall J., Cabré M., Serra-Prat M., Clavé P. (2010). Pathophysiology of oropharyngeal dysphagia in the frail elderly. Neurogastroenterol. Motil..

[6] Hanson B., O’Leary M.T., Smith C.H. (2012). The effect of saliva on the viscosity of thickened drinks. Dysphagia.

[7] Vallons K.J., Helmens H.J., Oudhuis A.A. (2015). Effect of human saliva on the consistency of thickened drinks for individuals with dysphagia. Int. J. Lang. Commun. Disord..

[8] Garcia J.M., Chambers E.t., Molander M. (2005). Thickened liquids: Practice patterns of speech-language pathologists. Am. J. Speech Lang. Pathol..

[9] Ortega O., Bolívar-Prados M., Arreola V., Nascimento W.V., Tomsen N., Gallegos C., Brito-de La Fuente E., Clavé P. (2020). Therapeutic Effect, Rheological Properties and α-Amylase Resistance of a New Mixed Starch and Xanthan Gum Thickener on Four Different Phenotypes of Patients with Oropharyngeal Dysphagia. Nutrients.

[10] Nsengiyumva E.M., Alexandridis P. (2022). Xanthan gum in aqueous solutions: Fundamentals and applications. Int. J. Biol. Macromol..

[11] Mortensen A., Aguilar F., Crebelli R., Di Domenico A., Frutos M.J., Galtier P., Gott D., Gundert-Remy U., Lambré C., Leblanc J.C. (2017). Re-evaluation of xanthan gum (E 415) as a food additive. EFSA J..

[12] Atkin J., Devaney C., Yoshimatsu Y., Smithard D. (2024). Modified medication use in dysphagia: The effect of thickener on drug bioavailability-a systematic review. Eur. Geriatr. Med..

[13] Tomita T., Goto H., Sumiya K., Yoshida T., Tanaka K., Kohda Y. (2016). Effects of Food Thickeners on the Inhibitory Effect of Voglibose Oral-disintegrating Tablets on Post-prandial Elevation of Blood Sugar Levels. Yakugaku Zasshi.

[14] Tomita T., Goto H., Sumiya K., Yoshida T., Tanaka K., Kudo K., Kohda Y. (2017). Effect of Food Thickener on the Inhibitory Effect of Mitiglinide Tablets on Post-prandial Elevation of Blood Glucose Levels. Dysphagia.

[15] Tomita T., Yamaguchi A., Nishimura N., Goto H., Sumiya K., Arakawa R., Yoshida T., Tachiki H., Kohda Y., Kudo K. (2019). Effect of food thickener and jelly wafer on the pharmacokinetics of levofloxacin orally disintegrating tablets. Heliyon.

[16] Blaszczyk A., Brandt N., Ashley J., Tuders N., Doles H., Stefanacci R.G. (2023). Crushed Tablet Administration for Patients with Dysphagia and Enteral Feeding: Challenges and Considerations. Drugs Aging.

[17] Takahashi N., Fujita Y., Takahashi N., Nakamura A., Harada T. (2020). Effect of xanthan gum-based food thickeners on the dissolution profile of fluoroquinolones oral formulations. J. Pharm. Health Care Sci..

[18] Zhang G.F., Liu X., Zhang S., Pan B., Liu M.L. (2018). Ciprofloxacin derivatives and their antibacterial activities. Eur. J. Med. Chem..

[19] Olivera M.E., Manzo R.H., Junginger H.E., Midha K.K., Shah V.P., Stavchansky S., Dressman J.B., Barends D.M. (2011). Biowaiver monographs for immediate release solid oral dosage forms: Ciprofloxacin hydrochloride. J. Pharm. Sci..

[20] Shariati A., Arshadi M., Khosrojerdi M.A., Abedinzadeh M., Ganjalishahi M., Maleki A., Heidary M., Khoshnood S. (2022). The resistance mechanisms of bacteria against ciprofloxacin and new approaches for enhancing the efficacy of this antibiotic. Front. Public Health.

[21] Forrest A., Nix D.E., Ballow C.H., Goss T.F., Birmingham M.C., Schentag J.J. (1993). Pharmacodynamics of intravenous ciprofloxacin in seriously ill patients. Antimicrob. Agents Chemother..

[22] Manrique Y.J., Lee D.J., Islam F., Nissen L.M., Cichero J.A., Stokes J.R., Steadman K.J. (2014). Crushed tablets: Does the administration of food vehicles and thickened fluids to aid medication swallowing alter drug release?. J. Pharm. Pharm. Sci..

[23] Logemann J.A., Gensler G., Robbins J., Lindblad A.S., Brandt D., Hind J.A., Kosek S., Dikeman K., Kazandjian M., Gramigna G.D. (2008). A randomized study of three interventions for aspiration of thin liquids in patients with dementia or Parkinson’s disease. J. Speech Lang. Hear. Res..

[24] Robbins J., Gensler G., Hind J., Logemann J.A., Lindblad A.S., Brandt D., Baum H., Lilienfeld D., Kosek S., Lundy D. (2008). Comparison of 2 interventions for liquid aspiration on pneumonia incidence: A randomized trial. Ann. Intern. Med..

[25] Parojcić J., Vasiljević D., Ibrić S., Djurić Z. (2008). Tablet disintegration and drug dissolution in viscous media: Paracetamol IR tablets. Int. J. Pharm..

[26] Parojcić J., Corrigan O.I. (2008). Rationale for ibuprofen co-administration with antacids: Potential interaction mechanisms affecting drug absorption. Eur. J. Pharm. Biopharm..

[27] Huupponen R., Seppälä P., Iisalo E. (1984). Effect of guar gum, a fibre preparation, on digoxin and penicillin absorption in man. Eur. J. Clin. Pharmacol..

[28] Harder S., Fuhr U., Beermann D., Staib A.H. (1990). Ciprofloxacin absorption in different regions of the human gastrointestinal tract. Investigations with the hf-capsule. Br. J. Clin. Pharmacol..

